# Oncological and functional outcomes of supratotal resection of *IDH1* wild-type glioblastoma based on ^11^C-methionine PET: a retrospective, single-center study

**DOI:** 10.1038/s41598-021-93986-z

**Published:** 2021-07-15

**Authors:** Seiichiro Hirono, Ko Ozaki, Masayoshi Kobayashi, Ayaka Hara, Tomohiro Yamaki, Tomoo Matsutani, Yasuo Iwadate

**Affiliations:** 1 Department of Neurological Surgery, Chiba University Graduate School of Medicine, 1-8-1 Inohana, Chuo-ku, Chiba-city, Chiba 260-8670 Japan; 2 Division of Nuclear Medicine, Chiba Ryogo Center, 3-30-1 Isobe, Mihama-ku, Chiba 261-0012 Japan

**Keywords:** Surgical oncology, Outcomes research, Medical research

## Abstract

The oncological and functional outcomes in glioblastoma (GBM) patients following supratotal resection (SupTR), involving complete resection of contrast-enhancing enhanced (CE) tumors and areas of methionine (Met) uptake on ^11^C-met positron emission tomography (Met-PET), are unknown. We conducted a retrospective review in newly diagnosed, *IDH1* wild-type GBM patients, comparing SupTR with gross total resection (GTR), in which only CE tumor tissue was resected. All patients underwent standard radiotherapy and temozolomide treatment, and were followed for tumor recurrence and overall survival (OS). Among the 30 patients included in this study, 7 underwent SupTR and 23 underwent GTR. Awake craniotomy with cortical and subcortical mapping was more frequently performed in the SupTR group than in the GTR group. During the follow-up period, significantly different patterns of disease progression were observed between groups. Although more than 80% of recurrences were local in the GTR group, all recurrences in the SupTR group were distant. Median OS in the GTR and SupTR groups was 18.5 months (95% confidence interval [CI] 14.2–35.1) and not reached (95% CI 30.5-not estimable), respectively; this difference was statistically significant (*p* = 0.03 by log-rank test). No postoperative neurocognitive decline was evident in patients who underwent SupTR. Compared to GTR alone, aggressive resection of both CE tumors and areas with Met uptake (SupTR) under awake craniotomy with functional mapping results in a survival benefit associated with better local control and neurocognitive preservation.

## Introduction

Glioblastoma multiforme (GBM) is one of the most malignant brain tumors. Maximal safe resection is the standard-of-care, first-line treatment strategy for patients newly diagnosed with GBM. Among known prognostic factors, including age, preoperative performance status, tumor location, and molecular profiles, extent of resection (EOR) is one of the most important prognostic factors for both low-grade glioma (LGG)^1,2^ and high-grade glioma (HGG)^3^. Furthermore, EOR is the only factor neurosurgeons can directly manipulate to improve the prognosis of patients with GBM. Since early surgical resection of LGG was found to be associated with better overall survival (OS) compared with biopsy and watchful waiting^4^, maximal safe resection of LGG with functional preservation has become the primary therapeutic strategy^5^. Recently, supratotal resection (SupTR) of LGG, which is defined as complete removal of any signal abnormalities in a volume of the postoperative cavity larger than the preoperative tumor volume^6^, was shown to be associated with prolonged time to malignant transformation^7^. With respect to newly diagnosed GBM, the standard goal of surgery has been gross total resection (GTR) of contrast-enhanced (CE) tumors on magnetic resonance imaging (MRI)^8^. However, recurrence of GBM is inevitable in the majority of patients who achieve GTR at initial surgery, primarily due to the highly infiltrative nature of this tumor type. Recently, several efforts to improve local disease control by safely expanding the resection margin beyond the CE area have been made. Beiko et al. first reported that maximal resection of both enhanced and nonenhanced disease (detectable on T1 noncontrast and T2/fluid-attenuated inversion recovery [FLAIR] MRI images) were associated with longer survival in select patients with malignant astrocytoma or GBM^9^. Following this study, several additional papers on this topic were published. Esquenazi confirmed the efficacy of SupTR for GBM^10^, which resulted in median OS of 54 months. However, neither molecular parameter nor neurocognitive outcome results have been reported. Li^11^ and Pessina^12^ presented their SupTR concept, in which the surrounding abnormal FLAIR region is resected if feasible and safe, reporting median OS of 20.7 months and 28.6 months, respectively. This extensive resection strategy and survival benefit were supported by the results of a retrospective multicenter cohort study^13^ that demonstrated a positive association between maximal resection of noncontrast tumors and OS in select GBM patients. However, it remains unclear whether SupTR is feasible and truly contributes to longer survival^14^, because no standardized definition of SupTR in the GBM surgery setting exists. Moreover, additional questions are raised. First, is there a subpopulation of GBM patients who are amenable to extensive resection? Second, is neuropsychological performance after SupTR truly feasible and acceptable? Unfortunately, detailed pre- and postoperative cognitive data are lacking in a majority of previous reports. Third, are T2/FLAIR images suitable for defining SupTR of GBM? MRI reveals structural abnormalities, and is not always suitable for distinguishing infiltrative tumors from vasogenic edema^15^.

The issues described above triggered us to evaluate the clinical role of metabolic imaging with positron emission tomography (PET) in SupTR of GBM. Methionine (Met), an essential amino acid, enables visualization of the metabolic activity of tumors via ^11^C-methionine PET (Met-PET), and is now successfully used in multiple GBM management settings, including diagnosis^16^, surgical planning^17^, radiotherapy^18^, and chemotherapy^19^. Therefore, combined aggressive resection of tissue that is both contrast-enhanced and demonstrates Met uptake in patients with GBM may provide a survival benefit. Concurrently, detailed neurocognitive assessment following removal of both gadolinium-enhanced tissue and tissue that demonstrates Met uptake should also be evaluated. Furthermore, *isocitrate dehydrogenase* (*IDH*) gene status must be included in patient selection criteria, as *IDH1*-mutated GBM has a unique natural history.

In this retrospective, single-center study, we aimed to clarify the possible survival benefit of additional resection of tissue demonstrating Met uptake beyond the contrast-enhanced region of tumors in patients with newly diagnosed, *IDH1* wild-type GBM, and to evaluate neuropsychological outcomes following SupTR.

## Methods

### Patient inclusion criteria, subgroups, and data collection

**Figure 1:**
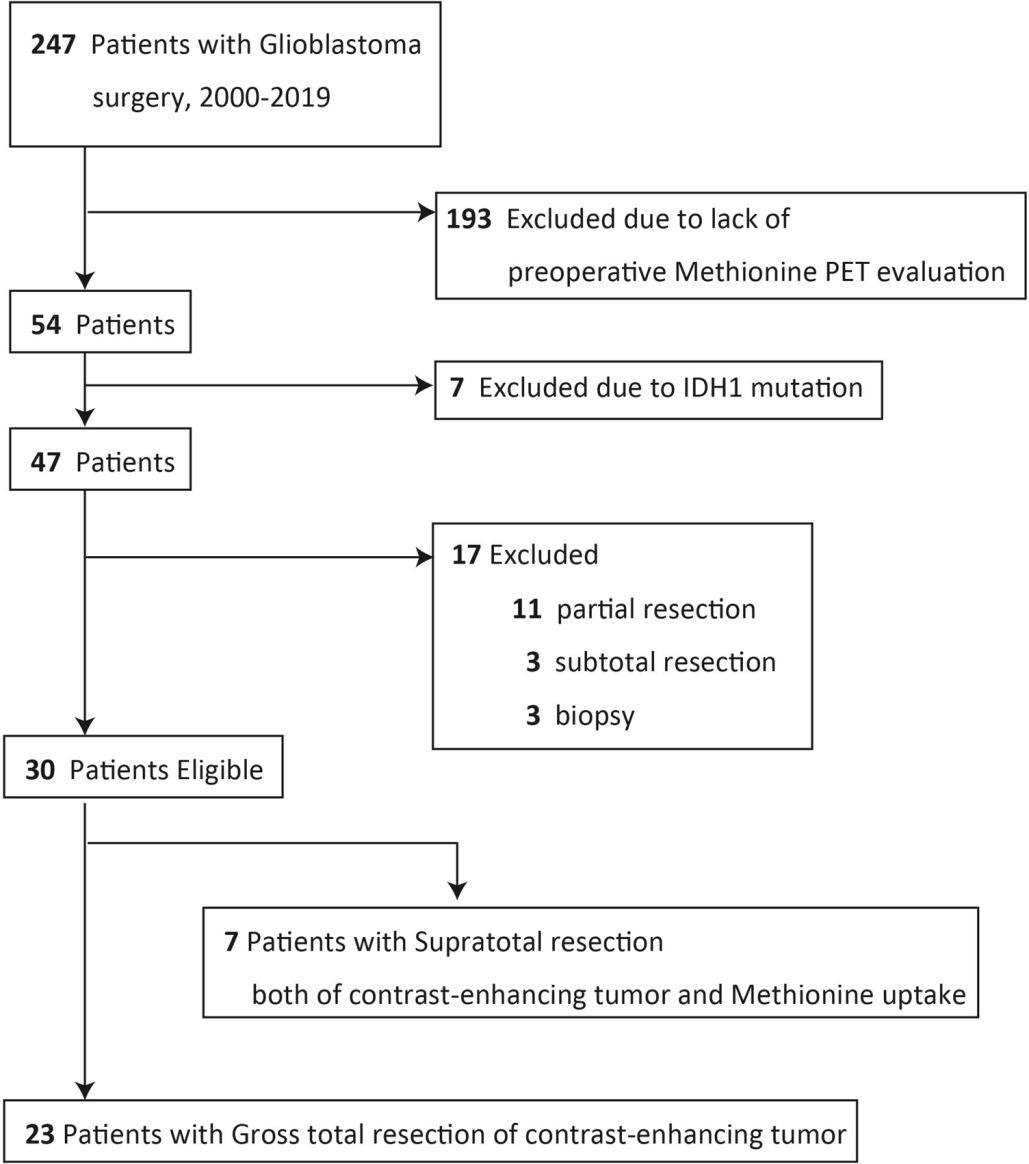
Data flow diagram for patient inclusion. Met-PET, ^11^C-methionine positron emission tomography; *IDH1*, *isocitrate dehydrogenase 1.*

All patients since 2000 with newly diagnosed and pathologically confirmed GBM who had undergone standard postoperative therapy^20^, which consisted of radiotherapy plus concomitant daily temozolomide (TMZ) followed by adjuvant TMZ, were retrospectively identified in our hospital records. Concomitant TMZ consisted of oral TMZ at a daily dose of 75 mg/m^2^ given 7 days per week from the first to the last day of radiotherapy, for a maximum of 49 days. After a 4-week break, patients received adjuvant TMZ (150–200 mg/m^2^) for 5 days every 28 days. MRI with contrast enhancement was repeated for all patients every 1 or 2 months after surgery as a routine clinical evaluation. Met-PET was also performed at the physician’s discretion as a part of clinical evaluation. Patients who satisfied the following four criteria were enrolled into this retrospective analysis (Fig. 1): (1) patients with newly diagnosed and pathologically confirmed GBM, (2) patients who underwent preoperative Met-PET evaluation, (3) patients with *IDH1* wild-type GBM, and (4) patients with no evidence of residual CE tumor on the immediate postoperative MRI performed within 24 to 72 h after surgical resection.

Among 247 GBM patients who were treated at our institution between 2000 and 2019, 193 patients were excluded due to lack of preoperative Met-PET evaluation. Next, 7 of 54 patients were excluded due to the presence of *IDH1* mutations. In addition, 17 patients were excluded because GTR of the enhanced lesion was not achieved. Finally, a total of 30 GBM patients were eligible and enrolled into this retrospective analysis. Volumetric analysis of the extent of resection for each patient was performed using a DICOM viewer OsiriX MD v.12.0.0 (Pixmeo, Bernex, Switzerland). GTR was defined as a lack of residual enhancement on postoperative imaging. These 30 patients were divided into two groups based on comparison of preoperative areas of Met uptake on Met-PET imaging and the resection cavity. Patients in whom complete resection of both CE areas and areas with Met uptake was achieved were categorized as the SupTR group. Patients in whom the resection cavity was larger than the CE area but smaller than the area of Met uptake were categorized as the GTR group. Patient characteristics and MRI images were retrieved from medical records through the end of November 2020. The Research Ethics Committees of the Graduate School of Medicine Chiba University approved this retrospective study (No. 3529, September 2019) and waived the need for informed consent for our retrospective study. The study was complied with all tenets of the Declaration of Helsinki.

### Surgery, MGMT, and IDH1 status

Surgical removal of tumors prior to radiotherapy plus concomitant daily TMZ was performed in all cases. In 12 out of 30 patients, awake craniotomy with cortical and subcortical direct electrical stimulation (DES) was performed at the neurosurgeon’s discretion for maximizing the resection and minimizing the postoperative deficit. The mapping methodology was based on Montpellier technique^21^ with a biphasic current (pulse frequency, 60 Hz; single pulse phase duration, 1 ms; amplitude, 2.0–4.5 mA). Methylation of the *O*^*6*^*-methylguanine-DNA methyltransferase* (*MGMT*) gene promoter was evaluated using methylation-specific polymerase chain reaction or immunohistochemically. *IDH1* mutation status was also immunohistochemically evaluated using the R132H mouse monoclonal antibody.

### Met-PET procedure and evaluation of uptake values

Met-PET scanning was performed at Chiba Ryogo Center using a Discovery MI (GE Healthcare, Tokyo, Japan) PET/computed tomography (CT) scanner with spatial resolution of 4.8 mm. Patients fasted for ≥ 6 h before PET scanning, and PET images were acquired with the patient in a resting state. Static scanning was performed for 6 min using 3-dimensional (3D) acquisition, and attenuation-corrected PET images were reconstructed using CT data by means of a 3D-ordered subset expectation maximization algorithm (20 subsets and 2 iterations). A Met dose of 370 MBq was injected intravenously within 1 min, with the scan starting 10 min after Met injection. Summation images covering 20 to 40 min after injection were used for analysis. Met uptake was semiquantitatively evaluated using the ratio T_max_/N_ave_, which is generated by dividing the maximum pixel count of the standardized uptake value of the tumor by the mean uptake of the contralateral normal frontal-lobe grey matter, avoiding the region affected by the tumor.

### Patient follow-up, functional evaluation, and evaluation of recurrence

Routine clinical evaluation with CE MRI was performed in all patients every 1 or 2 months after surgery as a routine clinical evaluation. Recurrence was evaluated using the RANO criteria^22^. Local recurrence (LR) was defined as tumor progression around the original site of the tumor, while distant recurrence (DR) was defined as a noncontiguous region from the site where the tumor initially existed. Dissemination was defined as subarachnoid spread of the disease^23^. As the clinical practice in our institution, neurocognitive function was intensively assessed only in patients who had undergone awake craniotomy. Therefore, detailed functional outcome data for all three time points (preoperatively and 1 week and 3 to 6 months after the surgery) were available for only 7 patients in the SupTR group. Executive and attention function was evaluated with Digit Span Forward (DS-F) and the trail-making test Part A and B (TMT-A and TMT-B). The Stroop test was also used to assess attention function. Nonverbal memory was assessed using Digit Span Backward (DS-B). Language function was monitored with a fluency test (categorical and phonological). Reading the Mind in the Eyes Test (Eyes test) was used for social behavior function^24^. Each raw score was converted into Z standardized scores (mean = 0, standard deviation = 1) based on the published Japanese normative data. A Z score ≤ − 1.645 for the DS, fluency, and Eyes tests and ≥ 1.645 (⍺ = 0.10 for 2-tailed prediction) for the TMT-A, -B, and Stroop tests indicated functional impairment.

### Statistical analysis

All statistical analyses were performed using JMP 11.2.1 software (SAS Institute, Cary, NC). The Kruskal–Wallis test was used for one-way analysis of variance in neurocognitive functional outcome assessment.

## Results

### Patients

A total of 30 GBM patients who underwent preoperative Met-PET evaluation and in whom GTR was achieved were included in this study. After comparing the resection cavity of CE lesions and areas of Met uptake, 7 patients were categorized into the SupTR group and the remaining 23 into the GTR group (Fig. 1). Clinical and pathological characteristics of patients are summarized in Table 1. No significant differences were observed in age at surgery, tumor location, or preoperative volume of CE lesions. The median uptake value of Met (T_max_/N_ave_) was 5.6 (range, 2.8–10.1) in the GTR group and 6.1 (range, 4.0–9.3) in the SupTR group. *MGMT* methylation status was similar between groups. Although more patients in the SupTR group (3 out of 7) had undergone tumor-treating fields (TTFields) therapy than patients in the GTR group (2 out of 23), the difference was not statistically significant. Remarkably, all resection procedures in the SupTR group were performed under awake craniotomy with cortical and subcortical mapping. In contrast, tumors were resected under general anesthesia in most patients in the GTR group (78%) (*p* < 0.0001). The median follow-up period was 16.6 months in the GTR group and 28.1 months in the SupTR group (*p* = 0.82).

**Table 1:** Clinical characteristic of 30 newly diagnosed glioblastoma patients.

Factor	Total	Gross total resection group	Supratotal resection group	*p*-value
**No. of patients**	30	23	7	
Male/female ratio	13:17	11:12	2:5	0.66
Median T_max_/N_ave_ of preoperative Met-PET images (range)	5.3 (2.8–10.1)	5.6 (2.8–10.1)	6.1 (4.0–9.3)	0.35
Median age at surgery, years (range)	57 (19–78)	57 (19–78)	56 (42–76)	0.69
Side of tumor, right/left	13/17	11/12	2/5	0.36
**Location of tumor, n**				
Frontal	12	10	2	0.69
Temporal	5	4	1	
Other	13	9	4	
**Preoperative Karnofsky performance score, n (%)**				0.18
0–60	5 (16.7)	5 (21.7)	0	
70–100	25 (83.3)	18 (78.3)	7 (100)	
**Postperative Karnofsky performance score, n (%)**				0.31
0–60	3 (10)	3 (13)	0	
70–100	27 (90)	20 (87)	7 (100)	
Median volume of enhanced volume before resection, cc (range)	18.8 (0.3–101.4)	19.7 (0.3–101.4)	14.7 (8.9–40.0)	0.38
Median volume of enhanced volume after resection, cc (range)	0	0	0	–
Median volume of nonenhanced volume before resection, cc (range)	58.9 (8.7–185.9)	59.5 (8.7–185.9)	29.6 (19.5–82.4)	0.18
Median volume of nonenhanced volume after resection, cc (range)	28.0 (2.7–103.3)	33.8 (2.7–103.3)	12.2 (3.8–31.9)	0.06
No. of patients with methylated MGMT, n (%)	12 (40%)	4 (57%)	8 (35%)	0.29
Median follow-up period for surgery, months (range)	19.5 (4.6–123.8)	16.6 (4.6–123.8)	28.1 (13.1–36.7)	0.82
No. of patients treated with bevacizumab, n (%)	10 (33%)	9 (39%)	1 (14%)	0.22
No. of patients implanted with carmustine wafer, n(%)	5 (17%)	5 (22%)	0	0.18
No. of patients treated with TTFields therapy, n (%)	5 (17%)	2 (8.7%)	3 (43%)	0.07
No. of patients who underwent awake craniotomy, n (%)	12 (40%)	5 (22%)	7 (100%)	< 0.0001

*Met-PET* ^11^C-methionine positron emission tomography, *KPS* Karnofsky performance status, *MGMT* O^6^-methylguanine-DNA methyltransferase, *TTFields* tumor-treating fields. All statistical analyses in Tables 1 and 2 were performed using JMP 11.2.1 software (www.jmp.com , SAS Institute, Cary, NC).

### Illustrative case presentation of SupTR with Met-PET

**Figure 2:**
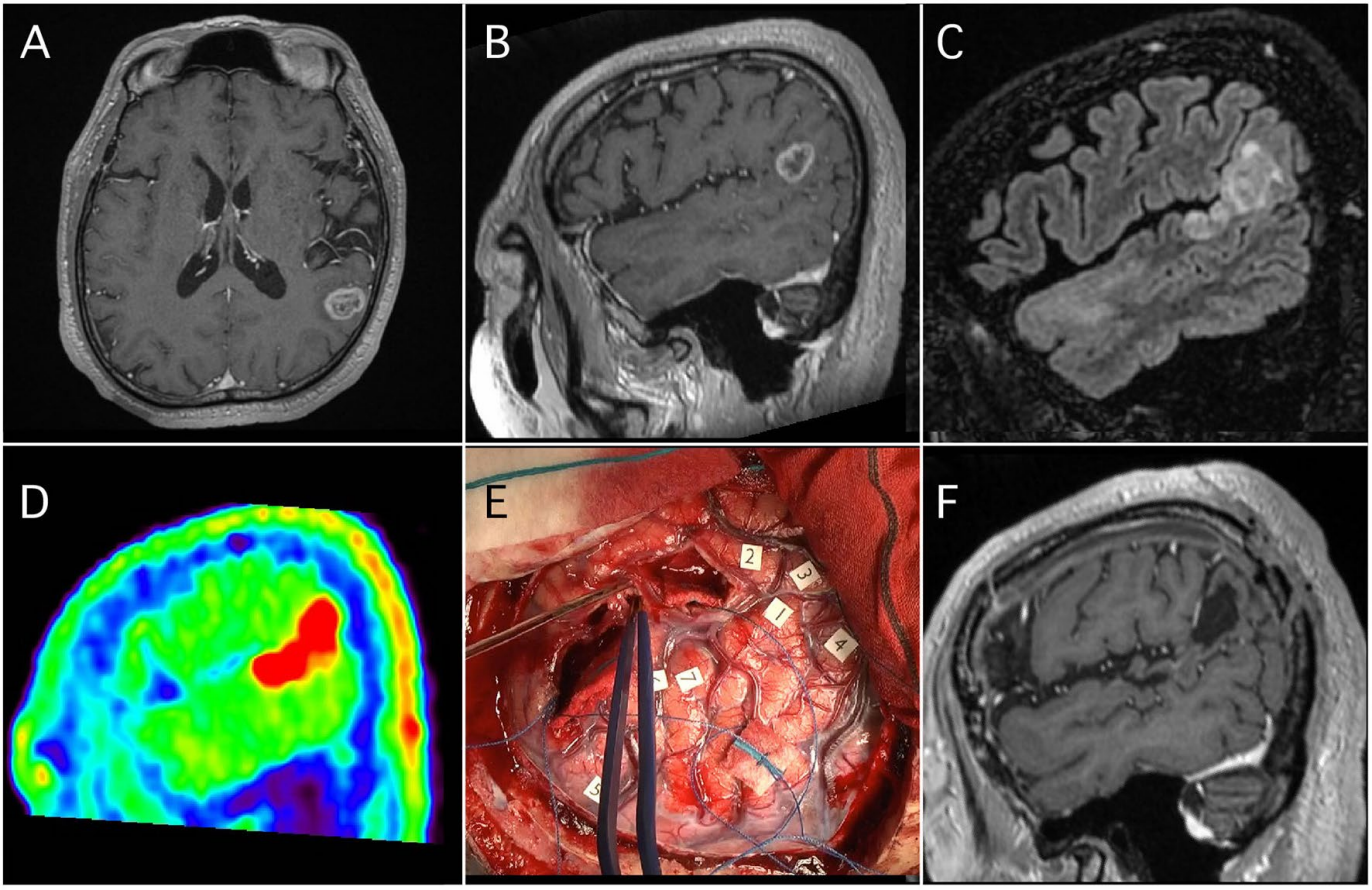
Illustrative case of supratotal resection with Met-PET. A contrast-enhanced tumor was observed in the left supramarginal gyrus (**A**,**B**). The abnormal FLAIR area was larger than the contrast-enhanced area (**C**), but smaller than the area of Met uptake (**D**) extending along the superior temporal gyrus. Supratotal resection of both the contrast-enhanced and area of Met uptake was achieved under awake cortical and subcortical mapping (**E**). At final follow-up imaging, neither LR nor DR was apparent (**F**).

**Figure 3:**
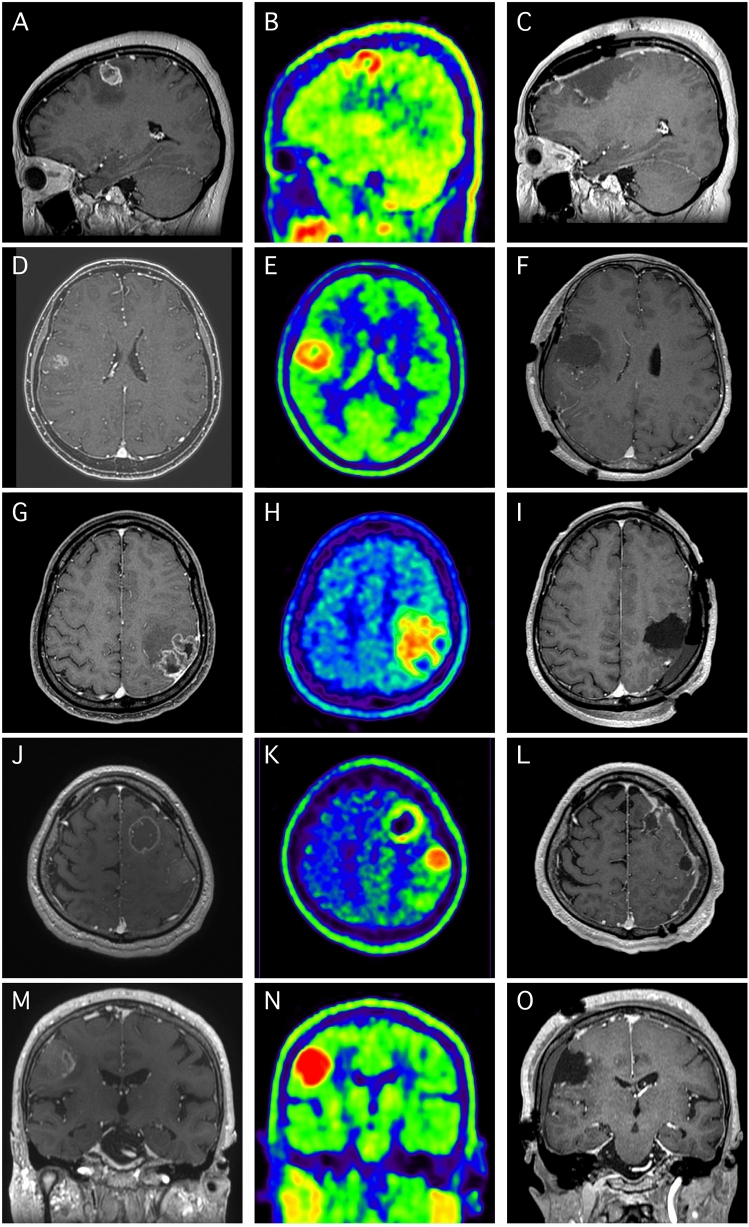
Preoperative MR and Met-PET images as well as postoperative MR images of remaining five patients in SupTR group. (**A**–**C**) A 47-year-old woman with seizure suffering from contrast-enhanced (CE) tumor in the left superior frontal gyrus (SFG) (**A**). The methionine uptake was observed in larger area than CE, extending anteriorly along the SFG (**B**). Both of CE and methionine area were removed under awake mapping. (**D**–**F**) A 56-year-old woman with dysesthesia in her left upper arm harboring small CE tumor in the right ventral postcentral gyrus (PostCG) (**D**). The Met accumulation was found in not only CE area but also surrounding subcortical white matter area (**E**). Supratotal resection (SupTR) of CE and Met uptake were achieved with awake craniotomy (**F**). (**G**–**I**) A CE tumor with subcortical low intensity area on MR images were demonstrated in a 42-year-old man (**G**). Note that Met uptake extend beyond the CE border into the subcortical area (**H**). SupTR of CE and Met uptake were accomplished with awake cortical and subcortical mapping (**I**). (**J**–**L**) A 59-year-old man with GBM in his left SFG (**J**). Met accumulation was multifocal in SFG, PostCG (**K**), and angular gyrus (not shown). All of CE lesion and Met uptake area were removed under awake surgery (**L**). (**M**–**O**) A 76-year-old woman with small CE lesion was found in her PostCG (**M**). Interestingly, Met uptake was observed in much larger area than CE lesion (**N**). SupTR, both of CE and Met uptake were obtained with awake craniotomy (**O**).

Figure 2 shows the radiological images of a 72-year-old, right-handed male patient in the SupTR group. A ring-enhanced lesion was observed in the left supramarginal gyrus (SMG) (Fig. 2A,B). The abnormal FLAIR area was larger than the CE area (Fig. 2C), but smaller than the area of Met uptake (Fig. 2D) extending along the superior temporal gyrus (STG). The T_max_/N_ave_ was 6.2. Both the CE lesion and area of Met uptake in the STG were completely removed under awake cortical and subcortical mapping (Fig. 2E) without any postoperative neurological deficit. The resection cavity and language deficit sites were close to each other. Radiotherapy plus concomitant daily TMZ followed by adjuvant monthly TMZ was continued. T1-weighted imaging with gadolinium 1 year after resection (Fig. 2F) showed excellent disease control without any tumor recurrence. More images of remaining six patients in SupTR group were available in Figs. 3 and 5.

### Functional outcomes following SupTR of GBM

**Figure 4:**
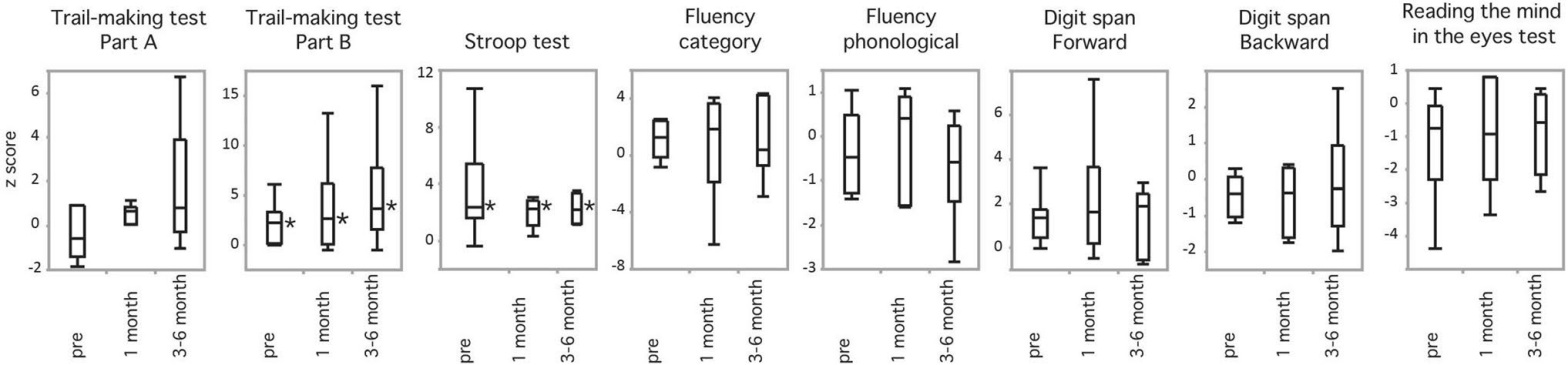
Box-plot representing median Z scores on eight functional batteries performed for 7 patients in the SupTR group preoperatively, 1 month after resection, and 3 to 6 months after resection. Asterisks indicate functional impairment if the median Z score ≤ − 1.645 on the DS, fluency, or Eyes tests or ≥ 1.645 on the TMT-A, B, or Stroop test.

The longitudinal results of eight neurocognitive batteries are summarized in Fig. 4. Since functional assessment was meticulously evaluated in patients who underwent awake craniotomy in our institution, these data were available for only 7 patients in the SupTR group. At preoperative baseline study, impairment (Z score ≥ 1.645 SDs above the normative data) using the TMT-B (Z score, 2.219) and Stroop test (Z score, 2.365) was observed, showing preoperative executive and attentional decline. However, preoperative Z scores for the other six tests were normal for language fluency, nonverbal memory, and social behavior function. After SupTR, TMT-B and Stroop test impairment that existed preoperatively continued to be observed at 1 month and at 3 to 6 months after surgery (Z score, 2.639 and 3.647 for TMT-B, respectively, and 2.274 and 2.175 for the Stroop test, respectively). In contrast, the other six batteries demonstrated no postoperative impairment at any time point. Additionally, no significant differences were observed for any battery across the three time points.

### Progression-free survival and tumor recurrence patterns

Table 2 shows details regarding disease control. During the follow-up period, disease progression or death from any cause occurred in 20 of 23 GTR patients (87%) and 4 of 7 SupTR patients (57%) (*p* = 0.08). Three patients in the GTR group died of pneumonia and other causes not related to progression of GBM. Interestingly, tumor recurrence patterns were remarkedly different between groups. In the GTR subgroup, more than 80% of cases of disease progression were characterized as LR. In contrast, all recurrences in the SupTR group were DR; the difference in recurrence patterns between groups was statistically significant (*p* < 0.001).

**Table 2:** Disease control and recurrence patterns.

Factor	Total n = 30	Gross total resection group (n = 23)	Supratotal resection group (n = 7)	*p*-value
No. of patients with disease progression or any cause of death, n (%)	24 (80)	20 (87)	4 (57)	0.08
**Recurrence pattern**				0.0004
Local recurrence, n (%)	15 (68)	14 (82)	0	
Distant recurrence, n (%)	4 (18)	1 (6)	4 (100)	
Leptomeningeal dissemination, n (%)	3 (14)	2 (12)	0	

### Illustrative case presentation of DR in the SupTR group

**Figure 5:**
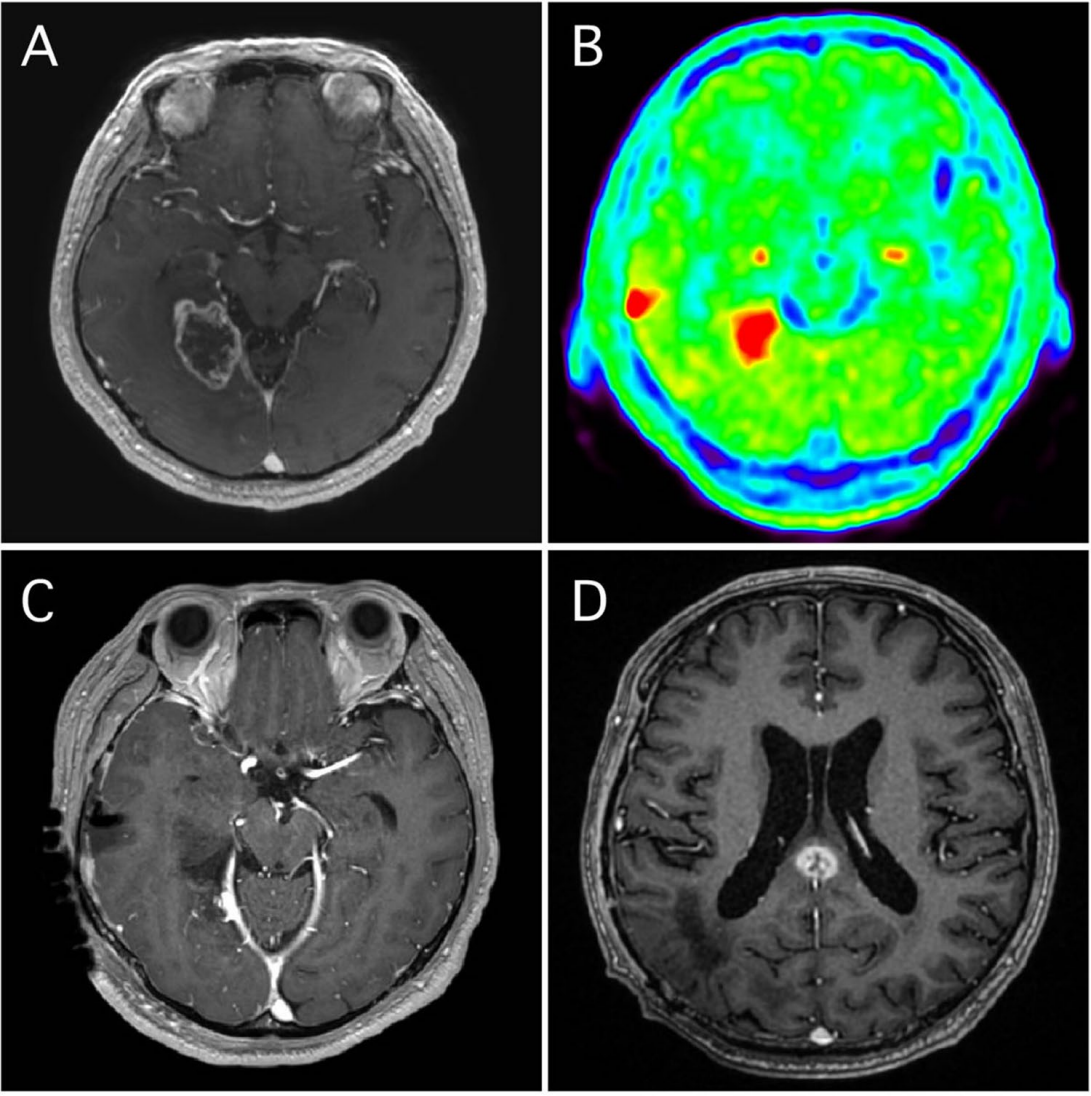
Illustrative case of distant recurrence after supratotal resection with Met-PET. A contrast-enhanced tumor was observed in the right parahippocampal and lingual gyri (**A**). Additionally, Met uptake was also evident in the right middle temporal gyrus (**B**) on PET. Supratotal resection of both the contrast-enhanced area and the area of Met uptake was completed under awake cortical and subcortical mapping for preservation of right optic radiation (**C**). However, distant tumor recurrence was demonstrated in the splenium of the corpus callosum 23.9 months after resection (**D**).

A representative case of DR after SupTR is shown in Fig. 5. The patient was a 52-year-old male whose original tumor was in the right parahippocampal and lingual gyri (Fig. 5A). A small but obvious lesion with T_max_/N_ave_ of 6.8 was also found in the right middle temporal gyrus (Fig. 5B) on Met-PET. The Met uptake value for the original tumor area was 5.2. Both lesions were completely resected in a single transcortical corridor under awake cortical and subcortical mapping used for preservation of optic radiation (Fig. 5C). Radiotherapy plus concomitant daily TMZ followed by adjuvant monthly TMZ was continued until a distant tumor recurrence was detected in the splenium (Fig. 5D). The original tumor site showed no evidence of LR at that time. The progression-free period was 23.9 months. The patient died of tumor progression 30.5 months after initial surgery.

### Overall survival

**Figure 6:**
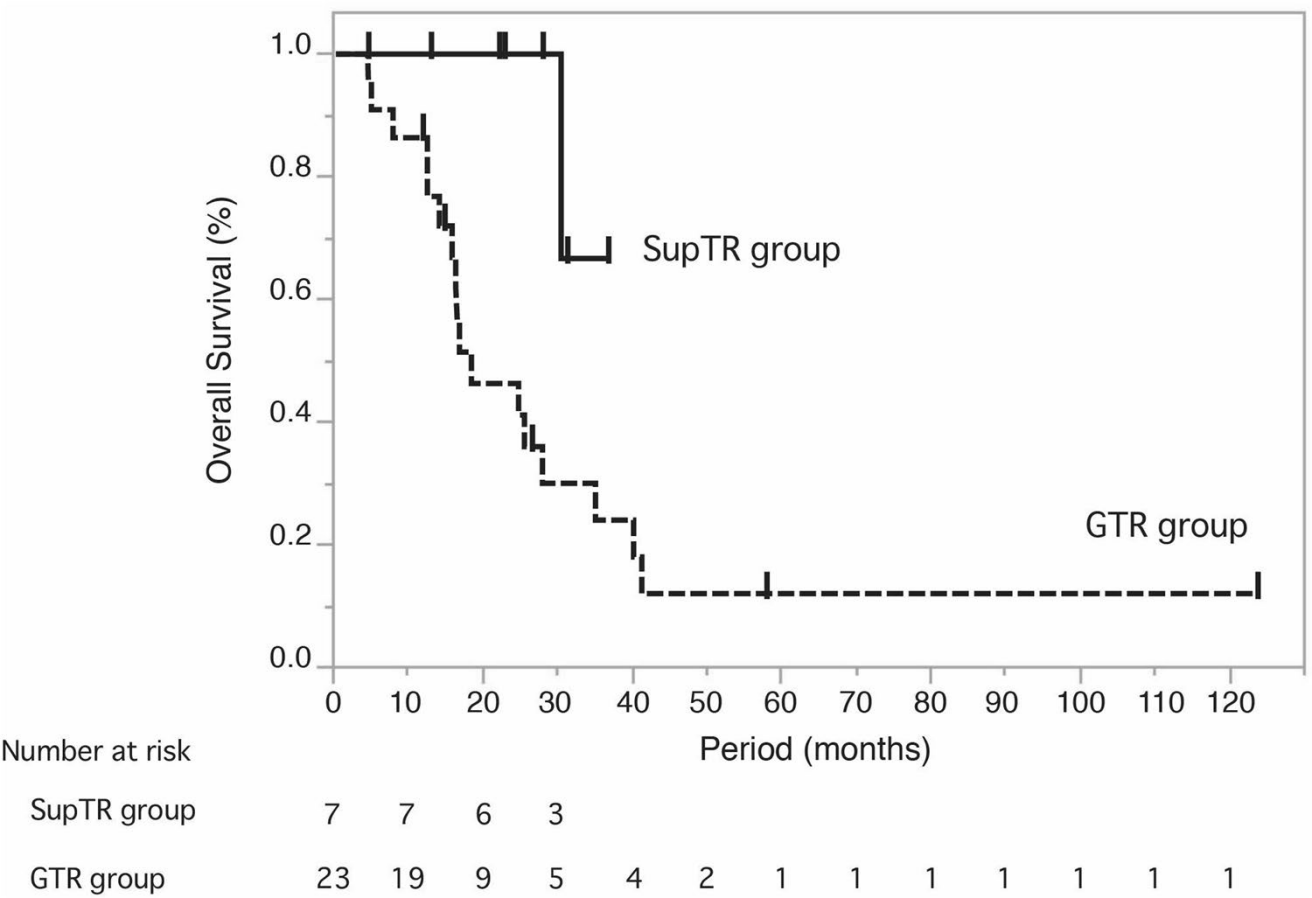
Kaplan–Meier curves for overall survival (OS) among SupTR patients (n = 7) and GTR patients (n = 23). Median OS was 18.5 months (95% confidence interval [CI] 14.2–35.1) in the GTR group, while median OS had not been reached (95% CI 30.5-not estimable) in the SupTR group. The estimated OS was significantly different between groups (*p* = 0.03 by log-rank test).

Kaplan–Meier curves illustrating OS in GTR and SupTR patients are shown in Fig. 6; median OS was 18.5 months in the GTR group (95% confidence interval [CI] 14.2–35.1), while median OS in the SupTR group had not been reached (95% CI 30.5-not estimable). The estimated OS rates were significantly different between groups (*p* = 0.03 by the log-rank test). The 1-year, 2-year, and 3-year OS rates were 86.4% (95% CI 5.2–95.5), 38.6% (95% CI 20.7–60.3), and 22.5% (95% CI 9.1–45.8), respectively, in the GTR group, and 100%, 100%, and 33.3% (95% CI, 4.3–84.6), respectively, in the SupTR group. The median follow-up period was 16.6 months (range, 4.6–123.8 months) in the GTR group and 28.1 months (range, 13.1–36.7 months) in the SupTR group (Table 1).

## Discussion

Today, the standard of care for newly diagnosed GBM consists of a combination of maximal safe resection with concomitant TMZ and radiotherapy followed by adjuvant monthly TMZ (the Stupp protocol^20^), which provides median OS of 12.7 to 21.7 months, depending on the methylation status of *MGMT*^25^. The addition of TTFields therapy, which changes electrical fields and interferes with mitotic tumor cells, has been demonstrated to extend OS in newly diagnosed GBM patients^26^. However, recurrence of GBM is inevitable in the majority of patients due to its aggressive infiltrative nature. The most common pattern of relapse, even after total resection of CE lesions, is LR^27^, which accounts for 80% of first instances of disease progression following treatment with the Stupp protocol. This strongly suggests that removal of CE areas alone is insufficient for local disease control. Therefore, we combined data from CE lesions using morphological MRI and accelerated metabolic information from Met-PET to evaluate the additional survival benefit of radical resection of CE areas and regions with Met uptake, which we define as SupTR in this study. We observed that longer OS was achieved using SupTR, which enabled better local control by reducing the incidence of LR, compared with simple GTR of CE lesions.

To date, many investigators have evaluated expanding the resection margin beyond the CE area. The concept of SupTR in glioma management was initially applied by Yordanova and Duffau^6^ in patients with LGG located in the so-called non-eloquent area. These patients underwent maximal safe resection under awake cortical and subcortical mapping of functional boundaries rather than anatomical boundaries, and reported better disease control and prevention of malignant transformation. Following this study, SupTR of GBM was further investigated primarily using the three different approaches described below.

Aldave et al.^28^ were the first group to report fluorescence-guided SupTR for GBM patients. These researchers defined SupTR as complete resection of both CE areas and 5-aminolevulinic acid (5-ALA) fluorescence-labeled tissue; this strategy provided median OS of 27.0 months in 25 patients. A similar study by Eyüpoglu^29^ et al. followed, in which resection was continued until the absence of any visible 5-ALA signal, resulting in median OS of 18.5 months in 30 patients. Considering the median OS of 18.8 months observed in the complete resection group of the Stupp protocol study^30^, integrated SupTR of CE and 5-ALA–labeled regions has provided a limited survival benefit. Moreover, functional outcomes following resection in these two studies were also limited.

A second type of SupTR was proposed by Li et al., who defined SupTR as total resection of CE lesions plus additional resection of the surrounding abnormal FLAIR region^11^. These authors reported that among GBM patients for whom the entire T1-weighted CE tumor was resected along with more than 53.21% of the abnormal FLAIR signal region, median OS of 20.7 months was achieved, suggesting a possible survival benefit. Pessina^12^ published similar results of prolonged survival following maximal resection beyond the CE margins and toward the boundaries of FLAIR abnormalities. They defined SupTR in more a radical way, as resection of 100% both of both CE and abnormal FLAIR areas. In their case series, SupTR was achieved in only 7.4% of the entire cohort, resulting in median OS of 28.6 months. Postoperative functional outcomes were limited to four simple categories: stable, improved, worsened, and new. These two reports focused on additional resection of abnormal FLAIR areas, with survival results similar to those associated with aggressive resection of 5-ALA–labeled areas^28,29^. In contrast, Mampre et al. retrospectively reviewed the relationship between the survival data and postoperative FLAIR volume in 245 GBM patients, and found no association between recurrence or survival among patients who underwent SupTR, with a median OS of 14.9 months^31^. A similar conclusion of lack of a survival benefit associated with FLAIR-based SupTR in a multicenter study was reported by Altieri et al^32^. Therefore, to our knowledge, more aggressive FLAIRectomy in GBM remains controversial. Given that the T2/FLAIR hyperintense areas surrounding tumors are either vasogenic edema or infiltrative tumor tissue, or both^15^, radical resection of nonenhanced areas is not expected to necessarily result in improved survival. Since routine MRI sequences, including T2/FLAIR and T1 CE images, are based on anatomical information, accurately distinguishing the true tumor from edematous tissue in surrounding nonenhanced areas is challenging.

We elected to use a different approach involving metabolic imaging with Met-PET, which enables visualization of active metabolic process occurring within GBM lesions. Currently, Met-PET is successfully used in surgical planning^17^, chemotherapy management^19^, and radiotherapy planning^18^ in GBM patients. Therefore, we focused on extensive resection of both CE areas and areas with Met uptake in patients with GBM. Our strategy is supported by Miwa et al.^33^, who reported discrepancies between areas with Met uptake and CE lesions in GBM patients, showing that areas of Met uptake are larger than CE areas in all cases. In addition, newly developed CE regions emerged in areas with initial Met uptake in 3 of 5 GBM patients in whom complete surgical resection of CE regions was achieved. These data further support our strategy of aggressive resection of areas of Met uptake along with CE lesions.

Recently, Müther and Stummer^34^ conducted a prospective observational single-center study evaluating the utility of postoperative 5-ALA fluorescence and uptake volumes on ^18^F-fluor-ethyl-tyrosine (^18^F-FET) PET for predicting survival. These researchers demonstrated that the postoperative ^18^F-FET-PET volume predicts OS, suggesting a survival benefit of SupTR guided by ^18^F-FET-PET. Patients with a residual ^18^F-FET-PET volume ≤ 4.3 cm^3^ experienced longer survival, with a predicted 2-year OS rate of 77.8% (95% CI 54.9–100.0). Both tyrosine and methionine are used as tracers in PET imaging, which provides higher diagnostic accuracy than anatomic MRI^35^; however, no data directly comparing these two tracers in surgical planning for GBM are available. Future studies of a possible survival benefit associated with additional resection of ^11^C-Met and ^18^F-FET areas are therefore required.

Interestingly, Beiko et al.^9^ reported that maximal surgical resection, including resection of both CE and non-CE areas, only contributed to improved survival in patients with *IDH1*-mutant malignant astrocytic tumors, but not *IDH1* wild-type tumors. It is worth addressing the SupTR-derived survival benefit for each molecular subgroup of GBM patients. However, no reliable methods are available to determine *IDH1* status preoperatively. Moreover, conventional MRI images of nonenhanced areas are less powerful for distinguishing true progressive tumor tissue, so it might be inadequate to perform resection based on those images considering both survival benefit and functional preservation. We excluded patients with *IDH1* mutations from this retrospective study (Fig. 1) to focus on the role of aggressive resection of areas with Met uptake. Evaluation of additional patients in a prospective study should be conducted to confirm the present results.

Consideration of neurocognitive outcomes is important for patients undergoing SupTR. Expanding resection margins may cause anatomical damage in nontumoral zones, leading to postoperative deficits. However, the majority of previous reports regarding SupTR have included limited^12,28,29^ functional outcome results. Herein we reported detailed functional outcomes using eight different cognitive batteries (Fig. 4), which revealed that no post-SupTR decline occurred following radical resection based on Met-PET results. Since GBM is one of the most highly infiltrative malignant brain tumors, neither lobectomy^36^ nor cerebral hemispherectomy^37^ can prevent tumor recurrence. Therefore, consideration of a balance between survival benefit and postoperative cognitive outcome is crucial. Currently, whether areas with Met uptake include normal parenchymal tissue has not been clarified. Fortunately, the 7 patients in the SupTR group demonstrated no abnormal responses during awake cortical and subcortical mapping inside the areas with Met uptake. Conversely, several abnormal responses were recorded in the surrounding normal brain following direct electrical stimulation.

In the present study, we demonstrated that more aggressive resection of GBM beyond CE areas using metabolic information provided by Met-PET can provide better local disease control and prolonged survival. However, this study has several limitations. The relatively small number of patients (n = 30) requires accumulation of additional data to validate the utility of SupTR with Met-PET in newly diagnosed GBM patients. This is primarily due to practical reasons; Met-PET evaluation is not covered by the public Japanese health insurance system. In addition, the lower spatial resolution of PET compared with MRI may also limit accurate delineation of ideal tumor resection areas. Finally, this study collected data retrospectively, thereby necessitating a prospective study to confirm the present results. However, our data clearly demonstrate a new role of Met-PET in GBM patients who undergo extended adjuvant TMZ therapy.

## Conclusion

The aggressive strategy of supratotal resection of both areas with gadolinium enhancement on MRI and Met uptake on Met-PET under awake cortical and subcortical mapping was demonstrated to be feasible and useful, and was associated with improved local control of GBM and a survival benefit in the absence of neurocognitive decline.
